# Interventions Addressing Vaccine Hesitancy in the WHO European Region and in North America (United States and Canada): A Systematic Review

**DOI:** 10.3389/phrs.2026.1609375

**Published:** 2026-04-09

**Authors:** Flavia Pennisi, Carlo Lunetti, Chiara Barbati, Luca Viviani, Anna Carole D’Amelio, Anabela da Conceição Pereira, Tiago Correia, Anna Odone, Carlo Signorelli

**Affiliations:** 1 Faculty of Medicine, Vita-Salute San Raffaele University, Milan, Italy; 2 PhD National Programme in One Health Approaches to Infectious Diseases and Life Science Research, Department of Public Health, Experimental and Forensic Medicine, Universita di Pavia, Pavia, Italy; 3 CIES-Iscte-IUL, Center for Research and Studies in Sociology, Iscte-University Institute of Lisbon, Lisbon, Portugal; 4 Global Health and Tropical Medicine, Associate Laboratory in Translation and Innovation Towards Global Health, Instituto de Higiene e Medicina Tropical, Universidade Nova de Lisboa, Lisbon, Portugal; 5 WHO Collaborating Center for Health Workforce Policies and Planning, Instituto de Higiene e Medicina Tropical (IHMT), Universidade Nova de Lisboa, Lisboa, Portugal; 6 Medical Direction, Fondazione IRCCS Policlinico San Matteo, Pavia, Italy

**Keywords:** Canada, healthcommunication, immunization programs, systematic review, United States

## Abstract

**Objective:**

Vaccine hesitancy threatens optimal immunization coverage. This review systematically identified and evaluated interventions addressing vaccine hesitancy in the WHO European Region and in North America (United States and Canada).

**Methods:**

A systematic search was conducted across PubMed, Scopus, PsycInfo, Cochrane Library, and Embase from inception to 17 January 2024. Eligible studies evaluated interventions targeting vaccine hesitancy. Data extraction and risk-of-bias assessment followed the methodological guidance of the Cochrane Handbook, and reporting adhered to PRISMA 2020 guidelines. The review protocol was registered in PROSPERO (CRD42024565588). Interventions were categorized as educational, communicational, policy-based, organizational, or digital.

**Results:**

A total of 59 studies met the inclusion criteria. Effective approaches included multicomponent strategies, community engagement, reminder and recall systems, educational campaigns, and legislative measures. Digital interventions yielded promising but heterogeneous results. The effectiveness of interventions was often enhanced when tailored to specific population needs and local contexts.

**Conclusion:**

Multifaceted interventions adapted to the sociocultural context appear most effective in reducing vaccine hesitancy in Europe and North America. Further high-quality studies are needed to refine implementation strategies and evaluate long-term impacts.

**Systematic Review Registration:**

https://www.crd.york.ac.uk/prospero/display_record.php?ID=CRD42024565588, identifier CRD42024565588.

## Introduction

Vaccine hesitancy is a barrier to achieving optimal vaccination coverage and public health safety. This complex issue is influenced by factors such as complacency, convenience, and confidence. Historically, events such as controversies over vaccine safety [1], changes in public health policies [2], and spread of misinformation [3, 4] have contributed to increased vaccine hesitancy. This term, ‘vaccine hesitancy’, describes the reluctance or refusal to vaccinate despite the availability of vaccines. These events underscore the dynamic nature of vaccine acceptance, which varies across time, place, populations and vaccines. Notably, in 2019, the World Health Organization (WHO) identified vaccine hesitancy as one of the greatest threats to global health [5].

The consequences of vaccine hesitancy are not merely theoretical but have manifested in tangible public health crises. The failure to maintain vaccination coverage has led to the resurgence of vaccine-preventable diseases in regions where they had previously been well controlled [6, 7]. For instance, in 2017 and again in 2024 [8], a significant measles outbreak occurred in Europe [9] and the United States [10] due to gaps in herd immunity, highlighting the critical need for sustained vaccination efforts [11]. Additionally, the 2014–2015 measles outbreak linked to Disneyland in California had a significant impact on public health policy [12].

Understanding the underlying factors of vaccine hesitancy and identifying effective strategies to counteract it are crucial for improving public health outcomes. Over the last decade, particularly following the COVID-19 pandemic, various interventions have been implemented in countries within the WHO European Region and in North America (United States and Canada) to address vaccine hesitancy [13]. These interventions include targeted health policies, education [14], information, and incentive campaigns [15]. However, there remains a pressing need to determine which of these strategies is most effective [16, 17].

This systematic review aims to evaluate the efficacy of various interventions designed to counter vaccine hesitancy across the WHO European Region and North America, focusing on studies conducted in the United States and Canada. By analysing interventions implemented in different socio-political contexts, the review seeks to extract key lessons and actionable insights to combat vaccine hesitancy and prevent the public health risks posed by disrupted vaccination programs, such as disease outbreaks and the resurgence of controlled diseases.

## Methods

The review was conducted according to the methodological guidance of the Cochrane Handbook for Systematic Reviews of Interventions [18] and reported following the Preferred Reporting Items for Systematic Reviews and Meta-Analyses (PRISMA 2020) statement [19]. The PRISMA 2020 checklist, indicating where each reporting item is addressed in the manuscript, is provided in Supplementary Table S1. A study protocol was registered with the International Prospective Register of Systematic Reviews (PROSPERO) under registration number CRD42024565588, registered on 4th July 2024.

### Search Strategy, Data Sources and Eligibility Criteria

The systematic review involved a comprehensive search across five databases, MEDLINE, Embase, Scopus, Cochrane Library, and PsycInfo, to identify studies on interventions aimed at reducing vaccine hesitancy. For the purpose of this review, Europe was operationally defined as the WHO European Region, while North America was limited to the United States and Canada, which were the countries included in the eligibility criteria. The search, updated on July 23, 2024, included studies with no temporal restrictions. The full search strategies for all databases are provided in Supplementary Table S1, including the complete search strings used for each database. The eligibility criteria for study inclusion were defined according to the PICOS framework (Population, Intervention, Comparators, Outcomes, and Study design/Setting). The population included community-based samples of the healthy general population, such as adults, parents, or mixed-age populations. Studies focusing exclusively on high-risk clinical groups (e.g., immunocompromised individuals, pregnant women, or people with specific chronic conditions) or narrowly defined professional subgroups (e.g., healthcare workers within a single institution) were excluded. This decision was made to avoid combining populations with substantially different baseline risks, healthcare pathways, and access to vaccination, thereby improving the external validity of the findings for the general population. The interventions considered were strategies aimed at reducing vaccine hesitancy or increasing vaccination uptake. The comparators included no intervention or alternative interventions when available. The outcomes of interest were vaccination uptake and vaccination intention. Eligible study designs included randomized controlled trials (RCTs), cohort studies, and controlled before–after studies. Only English-language articles published before July 4, 2024, were considered. A summary of the eligibility criteria according to the PICOS framework is provided in Supplementary Table S2.

### Data Collection, Extraction and Synthesis of Results

Results from the searches were exported as a Research Information Systems file (.ris) and imported into Rayyan, for title and abstract screening. Abstracts and full-text articles were independently screened by five reviewers (FP, CL, CB, LV and ACD) for eligibility. Any disagreements were resolved through discussion or through adjudication by a senior reviewer, if required. Data extraction was undertaken using a data extraction template by 5 authors (FP, CL, CB, LV and ACD), using Microsoft ExcelThe extracted data included information on authors, publication year, study characteristics (country, design, setting, target population and key sociodemographic features such as age distribution and education level when reported), interventions, control groups, and key results.

Studies were categorised by study type, study scale (local/regional vs. national), and vaccination type. The target populations were grouped into parents, adults, and the general population, which included different adult age groups like young adults (individuals aged 18–35) and the elderly (individuals aged over 75).

In line with widely used public health and behaviour change frameworks, we classified all interventions into four main categories: information-oriented, motivation-oriented, incentive-based, and mandatory interventions. This taxonomy reflects different primary mechanisms of action for changing vaccination behaviour, ranging from providing information and shaping attitudes to altering the external environment through incentives or requirements. Information-oriented interventions were defined as those primarily aimed at increasing knowledge or correcting misinformation about vaccines (e.g., educational materials, public information campaigns, factual web content). Motivation-oriented interventions were those designed to influence attitudes, emotions, social norms or personal commitment towards vaccination (e.g., motivational interviewing, narrative messages, tailored counselling, social norm feedback). Incentive-based interventions offered financial or non-financial rewards or removed costs to encourage vaccination (e.g., vouchers, monetary incentives, facilitated access or reminder programmes framed as benefits). Mandatory interventions relied on laws, regulations or organisational policies that made vaccination a requirement or attached consequences to non-vaccination (e.g., school-entry mandates, certification policies, employment requirements). Digital tools (e.g., websites, apps, SMS, social media, electronic health record prompts) were coded as delivery channels and could be used in any of the four categories; classification was based on the dominant content and mechanism of action rather than on the medium of delivery. This structured categorization facilitated clearer analysis and interpretation of the data, enabling a more effective evaluation of how different interventions impacted vaccine hesitancy and uptake.

Moreover, studies were stratified into two categories based on their scale: large-scale population-based studies and small-scale localized studies. Large-scale studies were defined as those involving interventions implemented at the national level, while small-scale studies focused on interventions conducted at regional or local levels.

### Outcome Measures and Analysis

For each included study, we extracted information on the outcome measurement instruments. For vaccination intention and related psychological constructs (e.g., hesitancy, trust, perceived risk), we recorded how outcomes were operationalised, including whether they were assessed with single items or multi-item questionnaires and any details reported on the response scale. When authors explicitly reported the use of validated instruments, this information was recorded. In particular, when available, we noted the use of validated scales commonly employed in vaccine hesitancy research, such as the Parent Attitudes about Childhood Vaccines (PACV) scale or other standardized questionnaires. However, several studies relied on single-item measures or study-specific instruments, contributing to heterogeneity in outcome assessment across studies.

For vaccination uptake, we extracted how the outcome was defined in each study as reported by the original authors. Studies were categorised as efficacious or inefficacious according to the conclusions reported by the authors of the included studies regarding the efficacy of the considered intervention on vaccination uptake or vaccination intention. For the purpose of synthesis, studies were classified as efficacious when the intervention produced a statistically significant improvement in vaccination uptake or vaccination intention (two-sided p < 0.05), or when the original authors reported a positive effect of the intervention on the outcome of interest. Studies reporting no statistically significant change or a negative effect were classified as inefficacious.

Where available in the original studies, we extracted effect size measures (e.g., mean differences, standardised mean differences) and any reported 95% confidence intervals. For studies that did not provide effect estimates or confidence intervals, we relied on the statistical tests or p-values reported by the authors to determine whether the intervention produced a statistically significant effect (two-sided p < 0.05). Because effect size reporting was inconsistent across studies and outcome definitions varied substantially, we did not perform additional effect size calculations or meta-analytic pooling.

### Risk of Bias

Risk of bias was assessed for all included studies using the Revised Cochrane Risk-of-Bias tool for randomized trials (RoB 2) and the Risk Of Bias In Non-randomized Studies of Interventions (ROBINS-I) tool for non-randomized studies. The assessment was conducted independently by two reviewers, and any disagreements were resolved through discussion or consultation with a third reviewer. The overall risk-of-bias judgment for each study was determined according to the criteria specified in the respective tools.

## Results

### Study Selection and Characteristics

**FIGURE 1:**
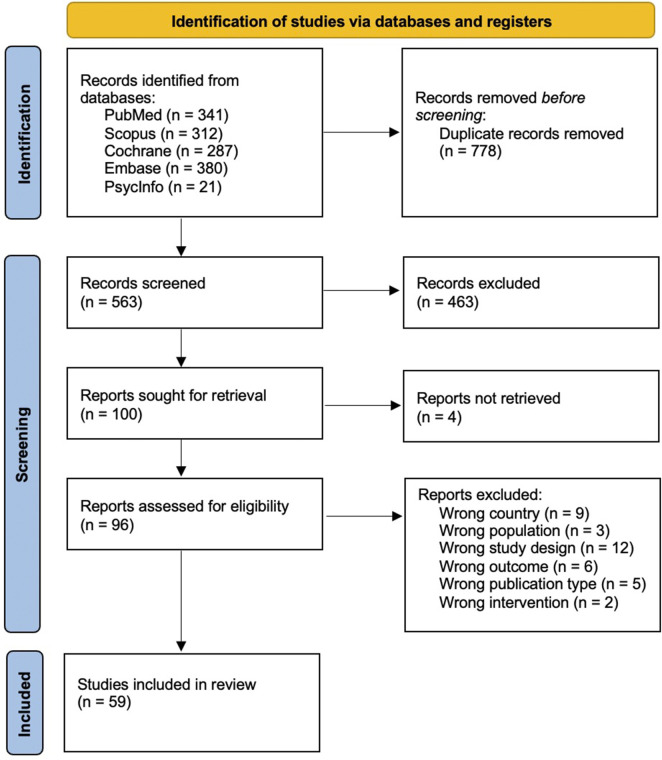
Preferred Reporting Items for Systematic Reviews and Meta-Analyses flow diagram of study search, screening, assessment and extraction. Interventions addressing vaccine hesitancy in the WHO European Region and in North America (United States and Canada): a Systematic Review (2013–2024).

The database searches identified a total of 1,341 studies, among which we selected 563 unique studies after removing duplicates. Following title and abstract screening, 463 papers were excluded. The initial screening process yielded 100 articles for further assessment against additional exclusion criteria. During the full-text screening stage, 37 articles were excluded for the following reasons: wrong country (n = 9), wrong population (n = 3), wrong study design (n = 12), wrong outcome (n = 6), wrong publication type (n = 5), and wrong intervention (n = 2). Finally, a total of 59 studies [20–50], [51–78] were included in the review. A PRISMA flowchart diagram illustrating the article selection process is presented in Figure 1.

**FIGURE 2:**
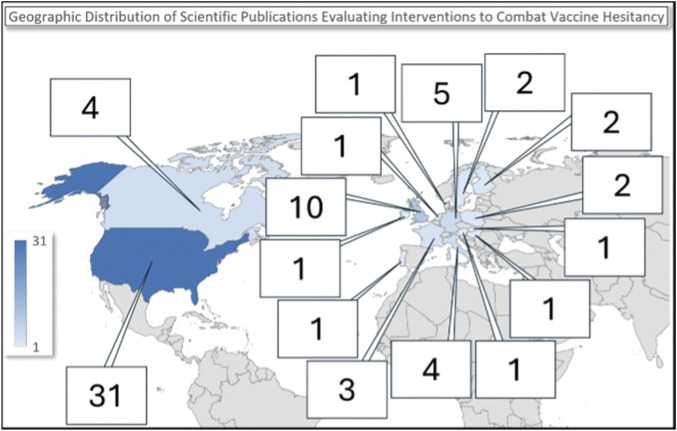
Map of interventions addressing vaccine hesitancy. Interventions addressing vaccine hesitancy in the WHO European Region and in North America (United States and Canada): a Systematic Review (2013–2024).

The systematic review encompassed studies published between 2013 and 2024 (Table 1). The period from 2021 to 2024 saw the highest number of publications, with 16 studies published in 2022 and 14 in 2023. The studies were conducted in North America (United States and Canada) (n = 34) [23, 25–28, 30–33, 35, 36, 39–42, 45, 46, 48, 51, 54–56, 58–60, 62, 66, 68, 69, 72, 74, 75, 77, 78] and in countries within the WHO European Region (n = 30) [20–22, 24, 29, 36, 38, 40, 44, 50, 52, 61, 63–65, 67, 70, 71, 73, 76], (Figure 2). The included studies were predominantly RCT (n = 46) [20–23, 26–31, 34, 35, 37–39, 41–43, 45, 47–49], [51–57, 59, 61, 62, 64–68, 70, 71, 73–79], followed by before-and-after study (n = 11) [24, 25, 32, 33, 36, 44, 46, 50, 58, 63, 72] and cohort study (n = 2) [40, 69].

**TABLE 1:** Study characteristics including authors, year of publication, journal, country, study design, study setting, vaccination type and target population. Interventions addressing vaccine hesitancy in the WHO European Region and in North America (United States and Canada): a Systematic Review (2013–2024).

Author	Year of publication	Journal	Country	Study setting	Vaccination target group	Target population category	Study design category
Altay S [20]	2021	Journal of experimental psychology applied	France	Large scale	Covid	Youngs	RCT
Batteux, E [34]	2022	BMJ open	UK	Small scale	Covid	Adults	RCT
Beleites F [34, 35]	2024	Internet interventions	USA	Small scale	Covid	Adults	RCT
Bender F. L [21]	2023	Health psychology	Germany	Large scale	Covid	General population	RCT
Betsch [22]	2015	The european journal of public health	Germany	Large scale	Pediatric vaccinations	Parents	RCT
Bialek M [36]	2023	Journal of experimental psychology: Applied	USA, UK, Poland, Portugal, other countries	Large scale	Respiratoy	Adults	Before-and-after study
Bradley-Ewing A [23]	2022	Human vaccines and immunotherapeutics	USA	Small scale	Respiratoy	Youngs	RCT
Burger M. N [24]	2022	PLOS ONE	Germany	Small scale	Others	General population	Before-and-after study
Buttenheim [37]	2020	Vaccine	USA	Large scale	Covid	Adults	RCT
Chiavenna C [38]	2023	Vaccine	Italy, UK	Small scale	Pediatric vaccinations	Parents	RCT
Cole J.W [25]	2022	Vaccine	USA	Small scale	Others	Parents	Before-and-after study
Cunningham R.M [26]	2021	HUMAN VACCINES and IMMUNOTHERAPEUTICS	USA	Small scale	Respiratoy	Adults	RCT
Dai H [27]	2021	Nature	USA	Large scale	Covid	Adults	RCT
Daley M.F [39]	2018	American journal of preventive medicine	USA	Large scale	Covid	Adults	RCT
					Respiratoy		
Debroy P [28]	2023	Health psychology	USA	Large scale	Others	Youngs	RCT
Eitze S [29]	2021	Health psychology	Germany	Large scale	Covid	General population	RCT
Fisher K.A [30]	2023	Vaccines	Canada	Large scale	Pediatric vaccinations	Adults	RCT
Fishman J [31]	2022	Vaccine	USA	Large scale	Covid	General population	RCT
Gagneur A [32]	2018	BMC public health	Canada	Large scale	Covid	Adults	Before-and-after study
Gagneur A [33]	2019	Eurosurveillance	Canada	Large scale	Respiratoy	Parents	Before-and-after study
Galasso V [40]	2023	BMJ global health	Australia, Austria, France, Germany, Italy, New Zealand, Sweden, the UK and the USA	Large scale	Covid	Adults	Cohort study
Glanz J.M [41]	2020	Pediatrics	USA	Large scale	Covid	Adults	RCT
Henrikson N.B [42]	2015	Pediatrics	USA	Large scale	Covid	Adults	RCT
Holford, D [43]	2024	Health psychology	UK	Large scale	Covid	Adults	RCT
Humlum, M.K [44]	2021	Medical decision making	Denmark	Large scale	HPV	Parents	Before-and-after study
Jacobson [45]	2022	Vaccine	USA	Large scale	Covid	Adults	RCT
Jamison K.C [46]	2022	Journal of pediatric nursing	USA	Large scale	Covid	Adults	Before-and-after study
Jimenez A.V [47]	2018	Social science and medicine	UK	Large scale	Covid	Adults	RCT
					Respiratoy		
Joslyn S [48]	2023	Journal of experimental psychology: Applied	USA	Large scale	Covid	General population	RCT
Kerr, J.R [49]	2021	Vaccines	UK	Large scale	Covid	Adults	RCT
La Torre, G [50]	2020	Vaccines	Italy	Small scale	Covid	Adults	Before-and-after study
Lewin, E.B [51]	2024	The journal of school nursing	USA	Large scale	Covid	Elderly	RCT
					Respiratoy		
Mäki K.O [52]	2023	PLOS ONE	Finland	Small scale	Pediatric vaccinations	Parents	RCT
Mills F [53]	2023	Vaccine: X	UK	Small scale	Respiratoy	General population	RCT
Opel D.J [54]	2019	Pediatrics	Washington	Large scale	Covid	Adults	RCT
Panozzo C.A [55]	2020	Journal of adolescent health	USA	Small scale	HPV	General population	RCT
Peters E [56]	2024	Journal of applied research in memory and Cognition	USA	Small scale	Others	Adults	RCT
Pfattheicher S [57]	2022	Health psychol	UK	Large scale	HPV	Youngs	RCT
Piltch-Loeb R [58]	2022	JMIR public health surveillance	USA	Small scale	Covid	Adults	Before-and-after study
Real F.J [59]	2017	Academic pediatrics	USA	Large scale	Covid	Adults	RCT
Reno J.E [60]	2019	Human vaccines and immunotherapeutics	Colorado	Large scale	Covid	Adults	RCT
Robertson D.A [61]	2022	Vaccine	Republic of Ireland	Small scale	Covid	Adults	RCT
Rodriguez R.M [62]	2023	JAMA internal medicine	USA	Small scale	Pediatric vaccinations	Adults	RCT
Ronzani P [63]	2022	Vaccine	Italy	Small scale	Pediatric vaccinations	Parents	Before-and-after study
Sääksvuori L [64]	2022	Plos medicine	Finland	Small scale	Others	Parents	RCT
Schneider F. H [65]	2023	Nature	Sweden	Small scale	Pediatric vaccinations	Parents	RCT
Sudharsanan N [66]	2022	eLife	USA and UK	Large scale	Others	Adults	RCT
Szaszi, A.J [67]	2024	Public health	Hungary	Small scale	Pediatric vaccinations	Youngs	RCT
Szilagyi P.G [68]	2023	Preventive medicine	USA	Large scale	Covid	Adults	RCT
Takagi M.A [69]	2023	Frontiers in public health	USA	Small scale	HPV	Parents	Cohort study
Teličák P [70]	2024	Applied Cognitive psychology	Slovakia	Large scale	Respiratoy	Elderly	RCT
Vandeweerdt C [71]	2022	Scientific reports	Denmark	Small scale	Pediatric vaccinations	Parents	RCT
Vaughn A.R [72]	2018	Vaccine	USA	Small scale	Pediatric vaccinations	Parents	Before-and-after study
Verger P [73]	2023	Euro surveill	France	Small scale	Covid	Adults	RCT
					Respiratoy		
Williams, S.E [74]	2013	Academic pediatrics	United States	Small scale	Others	Parents	RCT
Witus, L [75]	2022	PLOS ONE	USA	Large scale	Covid	General population	RCT
Yousuf H [76]	2021	EClinicalMedicine	Netherlands	Large scale	Covid	Adults	RCT
Zapf A.J [77]	2024	Patient education and Counseling	USA	Large scale	Covid	Adults	RCT
Zhu P [78]	2022	JMIR public health and surveillance	Canada	Large scale	Covid	Adults	RCT

**FIGURE 3:**
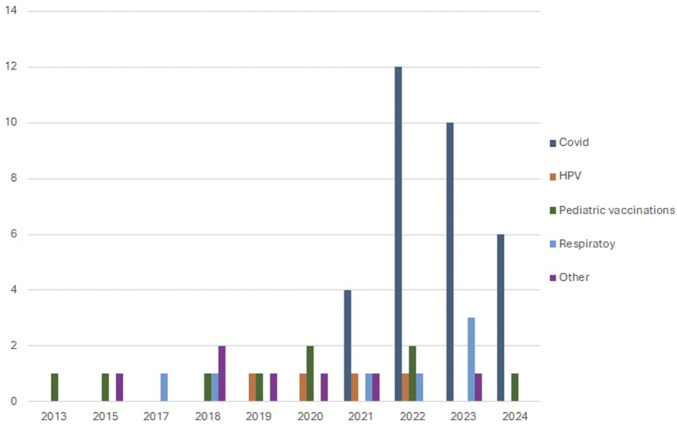
Distribution of vaccination target by year of publication among included studies. Interventions addressing vaccine hesitancy in the WHO European Region and in North America (United States and Canada): a Systematic Review (2013–2024).

COVID-19 immunizations were the predominant focus (n = 32 [20, 21, 27, 29, 31, 32, 34, 35, 37, 39–43, 45–51, 54, 58, 59, 61, 68, 73, 75–79], 45%), followed by pediatric vaccinations (n = 10 [22, 30, 38, 52, 62, 63, 65, 67, 71, 72], 16.7%), respiratory vaccines including influenza (n = 10 [23, 26, 32, 36, 39, 47, 51, 53, 70, 73], 11.7%), and Human Papillomavirus (HPV) vaccines (n = 4, 8.3%) [44, 55, 57, 69]. The remaining 7 studies (12%) [24, 25, 28, 56, 64, 66, 74] addressed other vaccine types (Figure 3).

Outcome measures and instruments varied across studies and are summarised in Tables 2, 3.

**TABLE 2:** Vaccination uptake: efficacy of interventions addressing vaccine hesitancy categorized by intervention type and study characteristics. Interventions addressing vaccine hesitancy in the WHO European Region and in North America (United States and Canada): a Systematic Review (2013–2024).

Author, year	Study design	Sample size (interventions and controls)	Intervention group	Channel	Vaccination uptake	Value	Evaluation metric employed	Value description
Altay S [20]	RCT	N = 614	Information-oriented	Web	Efficacious	ß = 0.12	Wave 1 questionnaire, three points-likert scale, wave 2 questionnaire	Regression coefficient
Bender F. L [21]	RCT	N = 652	Information-oriented	Video	Inefficacious	NA	Three items on a 6-point likert-type scale	NA
Betsch [22]	RCT	N = 297	Mandatory	Policy	Efficacious	NA	7 points scale	Pre-post comparison
Bradley-Ewing A [23]	RCT	N = 215	Motivation-oriented	Interview	Efficacious	(-10) - 3	Percentage	Intervention vs. control
Burger M. N [24]	Before-and-after study	N = 1,324	Information-oriented	Interview	Efficacious	NA	Scale (nd)	NA
Cole J.W [25]	Before-and-after study	N = 4,458	Information-oriented	Interview	Efficacious	11.5	Percentage	Intervention vs. control
Cunningham R.M [26]	RCT	N = 819	Motivation-oriented	Interview	Inefficacious	NA	Percentage	Intervention vs. control
Dai H [27]	RCT	NA	Incentives	Active call	Efficacious	1.06–6.07	Percentage	Intervention vs. control
Debroy P [28]	RCT	N = 28,941	Incentives	Web	Efficacious	1.5	Percentage	Intervention vs. control
Eitze S [29]	RCT	N = 585	Information-oriented	Web	Efficacious	NA	Scale (nd)	Intervention vs. control
Fisher K.A [30]	RCT	N = 615	Information-oriented	Interview	Efficacious	0.3	Percentage	Intervention vs. control
Fishman J [31]	RCT	N = 4,024	Incentives	Monetary	Efficacious	1.4–17.1	Percentage	Intervention vs. control
			Mandatory	Policy	Efficacious	1.4–8.6		
Gagneur A [32]	Before-and-after study	N = 2,389	Motivation-oriented	Text	Efficacious	0.15	Percentage	Pre-post comparison
Gagneur A [33]	Before-and-after study	N = 2,571	Motivation-oriented	Interview	Efficacious	12.3	Percentage	Pre-post comparison
Galasso V [40]	Cohort study	NA	Motivation-oriented	Text	Efficacious	2.25	Percentage	Intervention vs. control
Glanz J.M [41]	RCT	N = 824	Information-oriented	Web	Inefficacious	1.08	Odds ratio	Intervention vs. control
Holford, D [43]	RCT	N = 226	Motivation-oriented	Text	Efficacious	5.42	Scale (nd)	Intervention vs. control
Humlum, M.K [44]	Before-and-after study	N = 129,569	Motivation-oriented	Web	Efficacious	NA	NA	NA
Jacobson [45]	RCT	N = 2,701	Incentives	Monetary	Inefficacious	NA	Binary question	NA
Kerr, J.R [49]	RCT	N = 2,277	Information-oriented	Text	Inefficacious	NA	Scale (nd)	Intervention vs. control
Mäki K.O [52]	RCT	N = 1,099	Information-oriented	Web	Inefficacious	1.21	Percentage	Pre-post comparison
					Inefficacious	3.51	Percentage	Pre-post comparison
Mills F [53]	RCT	N = 2,726	Mandatory	Policy	Inefficacious	NA	Scale (nd)	NA
Opel D.J [54]	RCT	N = 156	Motivation-oriented	Interview	Inefficacious	NA	Percentage	Pre-post comparison
Panozzo C.A [55]	RCT	N = 762	Information-oriented	Video	Efficacious	0.4	10 points scale	Pre-post comparison
Peters E [56]	RCT	N = 1,284	Information-oriented	Web	Efficacious	0.03	6 points scale	Pre-post comparison
Real F.J [59]	RCT	N = 45	Information-oriented	Web	Efficacious	9.3	Percentage	Intervention vs. control
Reno J.E [60]	RCT	N = 187	Information-oriented	Interview	Inefficacious	NA	Percentage	Intervention vs. control
Robertson D.A [61]	RCT	N = 1,600	Motivation-oriented	Web	Inefficacious	NA	Percentage	Intervention vs. control
Rodriguez R.M [62]	RCT	N = 496	Information-oriented	Web	Efficacious	11.3	Percentage	Intervention vs. control
Ronzani P [63]	Before-and-after study	N = 2,277	Information-oriented	Web	Efficacious	1.6	Percentage	Intervention vs. control
Sääksvuori L [64]	RCT	N = 7,398	Incentives	Active call	Efficacious	NA	Percentage	NA
Schneider F. H [65]	RCT	N = 5,019	Incentives	Monetary	Efficacious	4	Percentage	Pre-post comparison
Szaszi, A.J [67]	RCT	N = 1,644	Motivation-oriented	Video	Inefficacious	NA	10 points scale	NA
Szilagyi P.G [68]	RCT	N = 213	Motivation-oriented	Web	Inefficacious	0.2	Percentage	Intervention vs. control
Takagi M.A [69, 70]	Cohort study	N = 234	Information-oriented	Video	Efficacious	0.2	Scale (nd)	Intervention vs. control
Teličák P [70]	RCT	N = 720	Information-oriented	Text	Efficacious	0.22	Scale (nd)	Intervention vs. control
Vandeweerdt C [71]	RCT	N = 222	Motivation-oriented	Web	Efficacious	5.8	10 points scale	Intervention vs. control
Verger P [73]	RCT	N = 733	Motivation-oriented	Interview	Efficacious	8	Percentage	Pre-post comparison
Williams, S.E [74]	RCT	N = 122	Information-oriented	Video	Inefficacious	NA	PACV scale	Intervention vs. control
Witus, L [75]	RCT	N = 1,632	Information-oriented	Video	Efficacious	NA	Odds ratio	NA
Yousuf H [76]	RCT	N = 980	Information-oriented	Video	Efficacious	0.11	5 point scale	Intervention vs. control
Zhu P [78]	RCT	N = 1,373	Motivation-oriented	Video	Efficacious	4.8	Percentage	Pre-post comparison

Abbreviations: NA, not applicable.

**TABLE 3:** Vaccination intention: efficacy of interventions addressing vaccine hesitancy categorized by intervention type and study characteristics. Interventions addressing vaccine hesitancy in the WHO European Region and in North America (United States and Canada): a Systematic Review (2013–2024).

Author, year	Study design	Sample size (interventions and controls)	Intervention group	Channel	Vaccination intention	Value	Evaluation metric employed	Value description
Batteux, E [34]	RCT	N = 328	Information-oriented	Video	Inefficacious	d = 0.34	5-Point likert scale	Pre-post comparison
Beleites F [35]	RCT	N = 792	Information-oriented	Video	Inefficacious	1.31	Questionnnaires	Intervention vs. control
			Motivation-oriented		NA	NA	List experiment	
Bender F. L [21]	RCT	N = 652	Information-oriented	Video	Inefficacious	NA	Three items on a 6-point likert-type scale	NA
Betsch [22]	RCT	N = 297	Mandatory	Policy	Inefficacious	−39	7 points scale	Pre-post comparison
Bialek M [36]	Before-and-after study	N = 1,191	Motivation-oriented	Interview	Efficacious	0.3–0.8	5-Point likert scale	Parallel group comparison
							Questionnnaires	
Burger M. N [24]	Before-and-after study	N = 1,324	Information-oriented	Interview	Inefficacious	NA	7 points likert scale	Intervention vs. control
Buttenheim [37]	RCT	N = 675	Information-oriented	Web	Inefficacious	NA	0–100 scale	Intervention vs. control
Chiavenna C [38]	RCT	N = 6,947	Information-oriented	Web	Efficacious	29.6	Probability scale: 0–100	Intervention vs. control
Daley M.F [39]	RCT	N = 945	Information-oriented	Web	Efficacious	0.12	5 point scale	Intervention vs. control
Gagneur A [32]	Before-and-after study	N = 2,571	Motivation-oriented	Interview	Efficacious	NA	Percentage	Pre-post comparison
Galasso V [40]	Cohort study	NA	Motivation-oriented	Text	Efficacious	NA	Probability scale: 0–10	Intervention vs. control
Henrikson N.B [42]	RCT	N = 347	Information-oriented	Video	Efficacious	2.3	PACV score	Pre-post comparison
Jacobson [45]	RCT	N = 2,701	Incentives	Monetary	Inefficacious	NA	Single question: 0–100 scale	NA
Jamison K.C [46]	Before-and-after study	NA	Motivation-oriented	Interview	Efficacious	2.60	Percentage	Intervention vs. control
Joslyn S [48]	RCT	N = 5,263	Information-oriented	Text	Efficacious	1.57	5-Point likert scale	Intervention vs. control
Kerr, J.R [49]	RCT	N = 2,277	Information-oriented	Text	Inefficacious	6.5	7 points likert scale	Intervention vs. control
La Torre, G [50]	Before-and-after study	N = 143	Information-oriented	Video	Efficacious	31.8	Percentage	Pre-post comparison
Lewin, E.B [51]	RCT	N = 47	Information-oriented	Web	Efficacious	1.9	Odds ratio	Intervention vs. control
Mills F [53]	RCT	N = 2,726	Mandatory	Policy	Inefficacious	NA	5-Point likert scale	NA
Pfattheicher S [57]	RCT	N = 2,005	Information-oriented	Web	Efficacious	0.24	Likert 7 points scale	Intervention vs. control
			Motivation-oriented		NA	0.30		​
Piltch-Loeb R [58]	Before-and-after study	N = 1991	Motivation-oriented	Video	Efficacious	0.28	5-Point likert scale	Intervention vs. control
			Information-oriented		Efficacious	0.28		Intervention vs. control
Reno J.E [60]	RCT	N = 187	Information-oriented	Interview	Inefficacious	NA	Percentage	Intervention vs. control
Robertson D.A [61]	RCT	N = 1,600	Motivation-oriented	Web	Inefficacious	NA	7 points likert scale	Intervention vs. control
Rodriguez R.M [62]	RCT	N = 496	Information-oriented	Web	Efficacious	11.9	Percentage	Intervention vs. control
Ronzani P [63]	Before-and-after study	N = 2,277	Information-oriented	Web	Efficacious	1.6	4 point likert scale	Intervention vs. control
Schneider F. H [65]	RCT	N = 5,019	Incentives	Monetary	Inefficacious	NA	7 points likert scale	Pre-post
Sudharsanan N [66]	RCT	N = 8,998	Information-oriented	Web	Efficacious	6.0	Percentage	Intervention vs. control
Szaszi, A.J [67]	RCT	N = 1,644	Motivation-oriented	Video	Inefficacious	NA	10 points likert scale	NA
Szilagyi P.G [68]	RCT	N = 213	Motivation-oriented	Web	Inefficacious	NA	Percentage	Intervention vs. control
Vaughn A.R [72]	Before-and-after study	N = 64	Information-oriented	Text	Efficacious	NA	NA	NA
Verger P [73]	RCT	N = 733	Motivation-oriented	Interview	Efficacious	33	Percentage	Pre-post comparison
Williams, S.E [74]	RCT	N = 122	Information-oriented	Video	Efficacious	6.7	PACV scale	Intervention vs. control
Zapf A.J [77]	RCT	N = 2,480	Information-oriented	Video	Efficacious	0.13	Percentage	Pre-post comparison
			Motivation-oriented		Inefficacious	−0.02		​
					Inefficacious	0.06		​

### Intervention Strategies and Target Populations

#### Large-Scale Interventions

This systematic review analyzed 35 [20, 22, 24, 29, 31, 34–38, 40, 42–44, 47–49, 52, 53, 55–59, 61, 63–67, 70, 75–78] national-scale studies aimed at assessing and improving vaccine uptake and reducing hesitancy on a large scale. The studies targeted diverse vaccination groups: COVID-19 (n = 26) [20, 24, 31, 34–36, 38, 40, 43, 48, 49, 52, 53, 56–58, 61, 63, 65–67, 70, 75–78], pediatric vaccinations (n = 2) [37, 42], HPV (n = 2) [44, 55], respiratory vaccines, such as influenza (n = 3) [29, 59, 64], and other vaccines (n = 2) [22, 42]. Sample sizes ranged from 500 to 20,000 participants. Adults were the primary target demographic (n = 23) [29, 34, 35, 37, 38, 40, 42, 48, 49, 52, 53, 55–58, 61, 63, 64, 66, 67, 70, 76, 78].

Interventions varied widely, with information-oriented interventions being most common (n = 24) [20, 24, 29, 34, 35, 37, 38, 41, 42, 47–49, 52, 55–59, 63, 66, 70, 75–77], including educational campaigns and public information dissemination through online platforms, community seminars, and workshops. Motivation-oriented campaigns (n = 11) [35, 36, 40, 43, 44, 57, 58, 61, 67, 77, 78] involved community engagement and the use of local leaders to promote vaccination. Three studies [31, 64, 65] employed incentive programs, offering financial or other rewards for vaccination, such as grocery vouchers or small financial incentives. Three studies [22, 31, 53] implemented mandatory vaccination policies, such as requiring vaccinations for school entry or employment.

#### Small-Scale Interventions

The review also included 24 [23–26, 34, 35, 38, 50, 52, 53, 55, 56, 58, 61–65, 67, 69, 71–74] small-scale studies, encompassing 16 local and 8 regional interventions. These studies addressed various vaccination groups such as COVID-19 (n = 6) [21, 27, 45, 62, 69, 71], respiratory vaccines (n = 3) [30, 67, 72], pediatric vaccinations (n = 8) (including Hepatitis B, Rotavirus, DTaP, IPV, Hib, and PCV) [25, 28, 32, 41, 46, 51, 54, 74] and HPV (n = 2) [23, 60] and 5 others [26, 32, 39, 50, 73]. Sample sizes for these small-scale studies varied, ranging from 100 to 5,000 participants, allowing for detailed, community-specific insights into vaccination challenges and successes.

The interventions employed diverse strategies, including information-oriented interventions (n = 12) [21, 25, 30, 39, 41, 50, 51, 60, 62, 69, 72, 74], with distribution of educational materials and small community meetings; motivation-oriented campaigns (n = 9) [23, 26, 32, 46, 54, 68, 71, 73], engaging local influencers and conducting targeted media campaigns; incentive programs (n = 3) [27, 28, 45] offering tangible rewards like gift cards or community recognition; digital and technological interventions (n = 7) [28, 39, 41, 51, 62, 68, 71], utilizing mobile health applications and electronic health record prompts for reminders and vaccination tracking.

### Efficacy of Vaccination Interventions

The efficacy of the various interventions is summarized in Tables 2, 3, which presents outcomes for vaccination uptake and vaccination intention. A substantial majority of studies (n = 44) [20, 23, 25, 27, 30–41, 43–46, 48, 50–53, 55–59], [62–64, 66, 67, 69, 70, 72–77, 80] reported statistically significant results.

#### Efficacy of Large-Scale Versus Small-Scale Interventions

To provide a comprehensive overview of intervention outcomes, we report both statistically significant and non-significant findings. While statistical significance strengthens the certainty of an effect, non-significant results still offer valuable insights into observed trends, whether positive or negative.

The efficacy of national interventions exhibited a range from modest to moderate improvements in vaccination uptake. Regarding vaccination uptake, efficacious interventions were reported in most studies (n = 17 [20, 22, 29, 31, 40, 43, 44, 55, 56, 59, 63–65, 70, 75, 76, 78]), while inefficacious outcomes were less frequently reported (n = 6 [24, 49, 52, 53, 61, 67]). The percentage differences between pre- and post-intervention measurements ranged from 1.2% to 17.1%. Concerning vaccination intention at the national level, efficacious interventions were reported in more than half of the studies (n = 11), whereas inefficacious interventions were noted in a substantial minority (n = 9). The percentage differences in hesitancy reduction varied from 2.3% to 29.6%, with the latter achieved through a web-based intervention.

Small-scale interventions demonstrated a broader range of efficacy. Improvements in vaccination uptake were reported in many studies (n = 11), with percentage increases ranging from 3% to 30%. A notable regional interview-based study reported a 30% increase in respiratory vaccination rates among the general population. Conversely, 8 studies [12, 60–66] concluded with inefficacious results). Regarding vaccination intention, most studies (n = 9 [55, 57, 58, 62, 67–71]) reported interventions that successfully reduced hesitancy, with reductions ranging from 2.6% to 33%. In contrast, a minority of studies (n = 4 [60, 61, 66, 72]) reported inefficacious interventions.

#### Efficacy Based on Intervention Type

The review identified varying efficacy across different intervention types (Tables 4, 5). Information-oriented interventions (n = 40, with 30 significant) were the most common, employing diverse channels such as web-based platforms (n = 16 [21–25, 35, 43–45, 49, 55, 63, 69, 70], with 15 significant), videos (n = 13 [19, 20, 32, 40–42, 48, 54, 61, 62, 68], with 10 significant), text-based communications (n = 5 [18, 34, 39, 67], with 3 significant), and interviews (n = 6 [33, 52, 53, 72], with 2 significant). These interventions showed improvements in vaccination rates ranging from 1.21% to 30%, with 13 studies reporting efficacious outcomes for vaccination uptake and 14 for reducing vaccine hesitancy.

**TABLE 4:** Efficacy of interventions on vaccination uptake by intervention type. Values correspond to the number of studies in each category. Interventions addressing vaccine hesitancy in the WHO European Region and in North America (United States and Canada): a Systematic Review (2013–2024).

Vaccination uptake	Efficacious	Inefficacious	Total
Incentives	5	1	6
Active call	2	​	2
Monetary	2	1	3
Web	1	​	1
Information-oriented	13	7	20
Interview	2	2	4
Text	1	1	2
Video	4	2	6
Web	6	2	8
Mandatory	2	1	3
Policy	2	1	3
Motivation-oriented	9	5	14
Interview	4	2	6
Text	2	​	2
Video	1	1	2
Web	2	2	4
Total	29	14	43

**TABLE 5:** Efficacy of interventions on vaccine hesitancy behaviour by intervention type. Values correspond to the number of studies in each category. Interventions addressing vaccine hesitancy in the WHO European Region and in North America (United States and Canada): a Systematic Review (2013–2024).

Vaccination intention	Efficacious	Inefficacious	Total
Incentives	​	2	2
Monetary	​	2	2
Information-oriented	14	6	20
Interview	​	2	2
Text	2	1	3
Video	5	2	7
Web	7	1	8
Mandatory	​	2	2
Policy	​	2	2
Motivation-oriented	6	3	9
Interview	4	​	4
Text	1	​	1
Video	1	1	2
Web	​	2	2
Total	20	13	33

Motivation-oriented campaigns (n = 23, with 16 significant) demonstrated promising results, utilizing various channels including interviews (n = 10 [46, 56–58, 64, 65, 71], with 8 significant), web-based platforms (n = 6 [30, 38, 59, 66], with 4 significant), videos (n = 4 [29, 37, 47], with 1 [51] significant), and text-based communications (n = 3 [27, 28], with 3 significant). These interventions showed 9 efficacious outcomes for vaccination uptake and 6 for reducing vaccine hesitancy.

Incentive programs (n = 8, with 5 significant) showed mixed results, with 5 studies reporting efficacious outcomes for vaccination uptake and one non efficacious. These interventions primarily used monetary incentives (n = 3 [17, 31, 60], with 3 significant), active calls (n = 2 [16, 50], with 2 significant) and 1 [36] efficacious web-based intervention. A notable example was a financial incentive of 200 SEK (∼$24) for the first dose of the COVID-19 vaccine. However, the impact on vaccine hesitancy was less positive, with no studies reporting efficacious outcomes and 2 [31, 60] reporting inefficacious results.

Mandatory interventions (n = 3, with 2 significant) demonstrated mixed efficacy. Three studies [17, 26, 36] reported efficacious outcomes for vaccination uptake, the same three studies showed a negative effect on vaccine hesitancy, with a significant decrease in intention to vaccinate in some cases. These interventions were primarily policy-based, including measures such as implementing COVID-19 certification policies across various settings. One study noted a considerable negative impact, with a mandatory policy leading to a 39% decrease in vaccine intention, highlighting potential challenges and unintended consequences of such approaches.

Of the included studies, 36 utilized technological interventions for vaccine promotion and addressing vaccine hesitancy. Video-based interventions were the most prevalent (n = 14, 38.9%), followed by internet-based interventions (n = 9, 25%), mobile health (mHealth) and SMS interventions (n = 6, 16.7%), social media-based interventions and online education programs/modules (n = 4 each, 11.1% each), Virtual Reality (VR) interventions (n = 2, 5.6%), and chatbot interventions, patient portal reminders, and gamified interventions (n = 1 each, 2.8% each). Video-based and internet-based interventions collectively accounted for 63.9% of the technological approaches identified. Regarding efficacy, the most efficacious interventions were video-based interventions, reporting increases in vaccination rates ranging from 10% to 20%. This was closely followed by mHealth applications, which yielded increases ranging from 10% to 18%, and online education programs/modules, which reported increases ranging from 10% to 17%. Internet-based interventions also showed notable efficacy, with increases ranging from 8% to 15%.

To facilitate comparison across intervention categories, a concise synthesis summarizing the number of efficacious and inefficacious studies and the approximate range of reported effects for each intervention type is presented in Supplementary Table S3.

#### Risk of Bias

The systematic review included a comprehensive evaluation of the risk of bias for all included studies, utilizing the Revised Cochrane Risk-of-Bias Tool for Randomised Trials (RoB 2) for 56 studies and the Risk of Bias in Non-randomised Studies of Interventions (ROBINS-I) tool for 3 studies. Overall, 14 studies (23.7%) were judged to have a low risk of bias, 24 (40.7%) were classified as having some concerns, and 21 (35.6%) were considered to have a high risk of bias. The distribution of overall risk-of-bias judgments across the included studies is illustrated in Supplementary Figure S1, while detailed results of the risk-of-bias assessment for each included study are reported in Supplementary Table S4.

## Discussion

### Summary of Main Findings and Interpretation

This systematic review provides information on the efficacy of interventions aimed at reducing vaccine hesitancy, offering an updated perspective on the topic previously outlined in several documents mostly published before the onset of the COVID-19 pandemic [81], and novel insights specifically focused on strategies for Western countries. We summarised evidence from 59 experimental studies covering a range of vaccination types, with a predominance of interventions related to COVID-19, followed by paediatric and respiratory vaccines. Main findings suggest that information- and motivation-oriented interventions were generally the most efficacious in improving participants' vaccination intentions and increasing vaccine uptake, compared with incentive- and mandatory interventions, which showed mixed or limited results. Furthermore, while no differences in efficacy were observed according to the type of vaccination targeted by the interventions, the increasing adoption of digital tools proved to be a promising strategy to tackle vaccine hesitancy, with strongest evidence in favour of video-based interventions and the rise of new approaches such as chatbots and virtual reality systems.

The geographical distribution of the included studies was predominantly concentrated in the USA and the UK, with most studies published recently. Different vaccination types were considered, with a major focus on SARS-CoV-2 vaccinations (45% of studies). The emphasis on SARS-CoV-2 related interventions is evidently caused by the urgent need for successful strategies aiding the widespread adoption of vaccines during the COVID-19 pandemic. This analysis also shows that the growth in number of publications seen during the 2020–2023 period was exclusively driven by research about SARS-CoV-2 vaccination, while the number of studies addressing the hesitancy for other vaccines remained relatively stable over the years.

The majority of the included studies focused on information-oriented and motivation-oriented campaigns, with generally positive results in terms of efficacy. These findings are consistent with a previous systematic review by Jarrett et al, which suggested that dialogue-based interventions, together with multi-component interventions, show better results in addressing vaccine hesitancy than incentive-based and reminder/recall-based interventions [81]. Among the included studies, Galasso et. al found that public health messages based on altruistic messages about protecting individuals, population health and the economy had significantly positive and sustained effects on increasing vaccination intentions, compared with message narratives about self-protection, which had no effect on vaccination uptake [40]. Another study, Zapf et al 2024, showed that receiving a fact-based intervention did not have a negative impact on vaccination intention, whereas narrative-based framing (i.e., information was provided via a story-telling format) appeared to have a negative impact [77]. In particular, it significantly reduced the intervention’s effectiveness compared to no framing (DiD = −0.15; 95% CI: −0.27 to −0.03; *p* = 0.014), possibly due to psychological reactance, where individuals perceive persuasive narratives as threats to their autonomy and thus resist the message [82]. Overall, our review found that both information-oriented and motivation-oriented approaches are efficacious, although we did not find sufficient evidence to determine whether one is more successful than the other in reducing vaccine hesitancy.

With regard to interventions based on incentive programmes, we found mixed results in terms of overcoming vaccine hesitancy. Four of the 6 included studies reported an increase in vaccine uptake. A notable example of this is represented by the study of Fishman et al., which examined various types of financial incentives concluding that guaranteed cash performed better than lottery-based incentives, and that the amount of money promised may be less important than the timeliness of the reward or the message framing when communicating about the incentives [31]. The validity of incentive-based interventions on vaccination uptake has been also reported by another recent systematic review [16], that identified this type of strategy as the most promising among all those evaluated. On the other hand, results about the impact of incentive-based interventions on vaccination intention are scarce, since only one of the 6 studies examined this outcome [65]. It’s interesting to note that this study shows that financial incentives don’t have an impact on vaccination intention both in the negative nor in the positive sense. This means that they are not reported to reduce vaccine hesitancy *per se*, but that they also do not affect the belief that vaccines are safe, nor people’s trust in researchers, the public health agency and pharmaceutical companies.

Finally, our findings indicate that mandatory vaccination appears to be the least efficacious strategy. Even though the number of studies using this strategy was also very low (only 3 out of 59), evidence suggested that neither vaccination intentions nor vaccination uptake improved after the implementation of this strategy. For example, the study performed by Betsch et al., reported that this type of strategy could be especially inefficacious toward individuals with a negative vaccination attitude, for whom vaccination uptake decreased by 39% after the institution of compulsory vaccination (compared to a decrement of 8% for individuals with a positive attitude towards vaccines) [22]. Although mandatory vaccination has historically proven to be effective in increasing vaccination coverage, some authors have argued that in the long term, vaccine mandates may have a negative impact on vaccination intentions and vaccine hesitancy, highlighting the importance of implementing behavioural and public trust promotion strategies [83, 84]. This is consistent with findings of another systematic review by Batteux et al. which suggested that the introduction of mandatory vaccination may have a negative impact, particularly on individuals' intention to vaccinate [34]. However, it should also be noted that, regardless of the type of approach used, the impact of the interventions on attitudes towards vaccination and vaccine uptake was found to be variable, due to differences in cultural contexts and countries of implementation, as well as the target group involved and their baseline vaccination intentions.

We also report that the majority of the included studies (36 out of 59) adopted digital modalities to conduct the interventions. In particular, our results show that video-based interventions were the most efficacious (10%–20% increases in vaccination uptake). Other successful digital interventions were conducted by the use of mHealth applications, online education programs/modules, and internet-based interventions. In a previous systematic review, text messaging, accessing immunisation campaign websites, web-based patient portals and computerised reminders were found to increase immunisation coverage, but there was insufficient evidence on the efficacy of using social networks, email communication and smartphone applications [85]. Meanwhile, in a subsequent study, we found that email-based reminder/recall interventions were efficacious in increasing vaccination coverage compared with no intervention, with potential for cost reduction, improved performance through automation, and effective outreach to target populations, although data for robust evidence of superiority over traditional methods were still limited [86]. Indeed, according to previous analysis, current adoption of digital technologies in immunisation programmes across Europe remains fragmented. Thus, identifying best practices and common standards for digital interventions to be successfully implemented in national vaccination strategies is necessary to support the need for effective lifelong immunisation services [87–89].

This study identified promising digital strategies to combat vaccine hesitancy, including the use of advanced technologies like chatbots and Virtual Reality. Altay et al. found that a chatbot providing information on COVID-19 vaccines significantly boosted vaccination intentions and improved attitudes toward vaccination [20]. Virtual reality was used in two studies. Real et al. created an immersive virtual reality curriculum to teach physicians communication skills when discussing vaccine hesitancy, resulting in a decrease in the rate of influenza vaccine refusal among patients being counselled in the period following the curriculum compared to the control group [59]. Vandeweerdt et al. investigated the effect of a first-person experience through immersive virtual reality on vaccination intentions; the motivational virtual reality intervention was almost three times more efficacious than communicating the same content through text and images [71].

National and local interventions to reduce vaccine hesitancy varied in scale, efficacy, and duration. Large-scale national strategies led to modest improvements in uptake (1.2%–17.1%) and reductions in avoidance (2.3%–29.6%). Small-scale interventions showed greater variability, with uptake increases from 3% to 30% and hesitancy reductions from 2.6% to 33%, with few reporting no effect. Interventions ranged from short-term (weeks/months, often for seasonal or emergency needs), to medium-term (months to a year, supporting community engagement for routine immunisations), to long-term (multi-year, for vaccines with multiple doses or targeting specific age groups, such as HPV).

### Limitations

This review has several limitations. Evidence was restricted to the WHO European Region and North America (United States and Canada) and studies targeting high-risk clinical groups were excluded, limiting the generalisability of findings. The general population itself was heterogeneous, and many studies lacked detailed sociodemographic data, preventing subgroup analyses. Considerable heterogeneity in interventions, settings, vaccines, outcomes and methods also precluded a quantitative meta-analysis. A consistent intention–behaviour gap emerged, with improvements in attitudes not always translating into higher uptake, likely due to structural and practical barriers. Most included studies were conducted during the COVID-19 period, where concurrent public health measures may have influenced hesitancy, reducing generalisability to other vaccines. Publication bias is possible, and relying on the term “vaccine hesitancy” in the search strategy may have missed relevant studies. In addition, the restriction to English-language publications and to specific study designs may have excluded relevant evidence from other contexts or methodological approaches.

Despite these limitations, this review provides one of the most comprehensive assessments of experimental interventions in Europe and North America. Future research should use standardised outcomes and validated measurement tools, broaden geographic and vaccine coverage, include subgroup analyses, and evaluate interventions in real-world contexts. Validated scales, such as the PACV and Vaccine Confidence Scale, are particularly important for accurately measuring changes in vaccine hesitancy [90]. Although this review focuses on Europe and North America, the identified strategies may provide useful insights for vaccination policies in other regions. However, the implementation of these interventions should be carefully adapted to local sociocultural contexts and health system characteristics.

### Conclusions

This review indicates that interventions reducing vaccine hesitancy in Europe and North America are most effective when they provide clear information or use motivational strategies, particularly through digital formats such as videos and web-based platforms. Across 59 experimental studies, these approaches improved vaccination uptake and intention, with increases of 1.2%–30% and reductions in hesitancy up to 33%. Video-based and mHealth interventions were especially effective. Mandatory measures sometimes increased uptake but often reduced intention, while incentives had limited impact on attitudes and only short-term effects on uptake. Overall, trust-building, context-specific strategies appear preferable to coercive approaches. Future work should use standardised measures, include a broader range of vaccines, and evaluate interventions in real-world settings.

## Data Availability

All data and materials relevant to this review are available within the article and its Supplementary Material. This includes the data extracted from the included studies, the data used for all analyses, and any other materials used in the review. No additional analytic code or external data collection templates were used beyond what is reported in the article and Supplementary Material.
