# Supplementary material for: Interventions Addressing Vaccine Hesitancy in the WHO European Region and in North America (United States and Canada): A Systematic Review
Source: Public Health Rev. 2026 Apr 9;47:1609375. doi: 10.3389/phrs.2026.1609375 (PMC13112373; doi:10.3389/phrs.2026.1609375)
Supplement: Supplementary file 1 [file Supplementaryfile1.docx]

# Supplementary file

Supplementary Table 1. Full database search strategies used for the systematic review. Interventions addressing vaccine hesitancy in the World Health Organization European Region and North America (United States and Canada), 2013–2024.

| **PUBMED:**  (“vaccination hesitancy”[Mesh] OR “vaccine hesitan*”[tiab] OR “vaccination hesitan*”[tiab] OR “vaccine-hesitan*” [tiab] OR “vaccination-hesitant*”[tiab]) **AND** (“intervention*”[tiab] OR “program*”[tiab] OR “polic*”[tiab] OR “campaign*”[tiab] OR “education*”[tiab] OR “promotion”[tiab] OR “awareness”[tiab] OR “practice”[tiab]) **AND**   ("randomized controlled trial"[Publication Type] OR "randomized controlled trials as Topic"[MeSH Terms] OR "non-randomized controlled trials as Topic"[MeSH Terms] OR RCT[Title/Abstract] OR RCTs[Title/Abstract]  OR "randomized control trial*"[Title/Abstract] OR "randomised control trial*"[Title/Abstract] OR experimental[Title/Abstract] OR trial*[Title/Abstract] OR quasiexperiment*[Title/Abstract] OR quasi-experiment*[Title/Abstract] OR non-randomized[Title/Abstract] OR non-randomised[Title/Abstract] OR “non randomized”[Title/Abstract] OR “non randomised”[Title/Abstract] OR nonrandomized[Title/Abstract] OR nonrandomised[Title/Abstract]) |
| --- |
| **SCOPUS:**  (INDEXTERMS("vaccination hesitancy") OR TITLE-ABS("vaccine hesitan*") OR TITLE-ABS("vaccination hesitan*") OR TITLE-ABS(vaccine-hesitan*) OR TITLE-ABS(vaccination-hesitant*)) AND (TITLE-ABS(intervention*) OR TITLE-ABS(program*) OR TITLE-ABS(polic*) OR TITLE-ABS(campaign*) OR TITLE-ABS(education*) OR TITLE-ABS(promotion) OR TITLE-ABS(awareness) OR TITLE-ABS(practice)) AND (INDEXTERMS("randomized controlled trials") OR INDEXTERMS("non-randomized controlled trials") OR TITLE-ABS(RCT) OR TITLE-ABS(RCTs) OR TITLE-ABS("randomized control trial*") OR TITLE-ABS("randomised control trial*") OR TITLE-ABS(experimental) OR TITLE-ABS(trial*) OR TITLE-ABS(quasiexperiment*) OR TITLE-ABS(quasi-experiment*) OR TITLE-ABS(non-randomized) OR TITLE-ABS(non-randomised) OR TITLE-ABS("non randomized") OR TITLE-ABS("non randomised") OR TITLE-ABS(nonrandomized) OR TITLE-ABS(nonrandomised)) |
| **COCHRANE:**  ([mh "vaccination hesitancy"] OR ("vaccine" NEXT hesitan*):ti,ab OR ("vaccination" NEXT hesitan*):ti,ab OR vaccine-hesitan*:ti,ab OR vaccination-hesitant*:ti,ab) AND (intervention*:ti,ab OR program*:ti,ab OR polic*:ti,ab OR campaign*:ti,ab OR education*:ti,ab OR promotion:ti,ab OR awareness:ti,ab OR practice:ti,ab) AND  ("randomized controlled trial":pt OR [mh "randomized controlled trials as Topic"] OR [mh "non-randomized controlled trials as Topic"] OR RCT:ti,ab OR RCTs:ti,ab OR ("randomized control" NEXT trial*):ti,ab OR ("randomised control" NEXT trial*):ti,ab OR experimental:ti,ab OR trial*:ti,ab OR quasiexperiment*:ti,ab OR quasi-experiment*:ti,ab OR non-randomized:ti,ab OR non-randomised:ti,ab OR "non randomized":ti,ab OR "non randomised":ti,ab OR nonrandomized:ti,ab OR nonrandomised:ti,ab) |
| **EMBASE:**  ('vaccination hesitancy'/exp OR 'vaccine hesitan*':ti,ab OR 'vaccination hesitan*':ti,ab OR vaccine-hesitan*:ti,ab OR vaccination-hesitant*:ti,ab) AND (intervention*:ti,ab OR program*:ti,ab OR polic*:ti,ab OR campaign*:ti,ab OR education*:ti,ab OR promotion:ti,ab OR awareness:ti,ab OR practice:ti,ab) AND ('randomized controlled trials'/exp OR 'non-randomized controlled trials'/exp OR RCT:ti,ab OR RCTs:ti,ab OR 'randomized control trial*':ti,ab OR 'randomised control trial*':ti,ab OR experimental:ti,ab OR trial*:ti,ab OR quasiexperiment*:ti,ab OR quasi-experiment*:ti,ab OR non-randomized:ti,ab OR non-randomised:ti,ab OR 'non randomized':ti,ab OR 'non randomised':ti,ab OR nonrandomized:ti,ab OR nonrandomised:ti,ab) |
| **PSYCHINFO:**  (("vaccination hesitancy" OR vaccine NEXT hesitan* OR vaccination NEXT hesitan* OR vaccine-hesitan* OR vaccination-hesitant*) AND (intervention* OR program* OR polic* OR campaign* OR education* OR promotion OR awareness OR practice)) AND (("randomized controlled trial" OR "randomized controlled trials as Topic" OR "non-randomized controlled trials as Topic" OR RCT OR RCTs OR "randomized control trial*" OR "randomised control trial*" OR experimental OR trial* OR quasiexperiment* OR "quasi-experiment*" OR non-randomized OR "non-randomised" OR "non randomized" OR "non randomised" OR nonrandomized OR nonrandomised)) |

Supplementary Table 2.  A priori defined inclusion and exclusion criteria according to the Population, Intervention, Comparator, Outcomes and Study design framework. Interventions addressing vaccine hesitancy in the World Health Organization European Region and North America (United States and Canada), 2013–2024.

| Search strategy | Details |
| --- | --- |
| Inclusion criteria | **P**: Healthy general population, with no age limits. Elderly individuals, even those with comorbid conditions, were included. **I**: Any type of intervention to counter vaccine hesitancy for any type of vaccination in WHO European Region and North America (USA and Canada) **C**: Absence of intervention or alternative intervention  **O**: Any quantitative outcome assessing the effectiveness of interventions to counter vaccine hesitancy, including vaccination knowledge and vaccine uptake (assessed through two key outcome measures: vaccination uptake and changes in vaccination intention, typically evaluated through standardized scales or questionnaires that assess individuals' beliefs, intentions, and confidence regarding vaccines. **S**: Experimental studies |
| Exclusion criteria | Observational studies, thesis, protocols, books, book chapters, studies not published as peer-reviewed, no full-text papers (abstract, conference paper, letter, commentary, erratum, correction, editorial, note), qualitative studies and reviews. Non-English language articles.  Excluded from this review are all studies that narrow their focus to specific subgroups which may introduce bias into the assessment of vaccine hesitancy. This includes studies exclusively targeting chronic illness patients, pregnant women, healthcare workers, and socio-economic subgroups. |
| Language filter | English |
| Time filter | No time filters |
| Database | MEDLINE, Embase, Scopus, Cochrane Library, PsycInfo |

Supplementary Table 3. Summary of intervention effectiveness according to intervention category. Interventions addressing vaccine hesitancy in the World Health Organization European Region and North America (United States and Canada), 2013–2024.

| **Intervention category** | **Total studies** | **Efficacious** | **Inefficacious** | **Approximate effect range reported** |
| --- | --- | --- | --- | --- |
| Information-oriented | 20 | 13 | 7 | 1–30% increase in vaccination uptake or intention |
| Motivation-oriented | 14 | 9 | 5 | 3–33% increase in vaccination uptake or intention |
| Incentive-based | 6 | 5 | 1 | 1–17% increase in vaccination uptake |
| Mandatory | 3 | 2 | 1 | Mixed effects; some studies reported decreases in vaccination intention |

Supplementary Table 4. Risk of bias assessment of the included studies. Interventions addressing vaccine hesitancy in the World Health Organization European Region and North America (United States and Canada), 2013–2024.

| **Study** | **Year** | **Study design category** | **Risk of bias tool** | **Overall risk of bias** |
| --- | --- | --- | --- | --- |
| Altay S. | 2021 | RCT | RoB 2 | Some concerns |
| Batteux, E. | 2022 | RCT | RoB 2 | Some concerns |
| Beleites F. | 2024 | RCT | RoB 2 | Low risk |
| Bender F. L. | 2023 | RCT | RoB 2 | Some concerns |
| Betsch | 2015 | RCT | RoB 2 | Some concerns |
| Bialek M. | 2023 | Before–after / non-randomized study | ROBINS-I | Moderate |
| Bradley-Ewing A. | 2022 | RCT | RoB 2 | Low risk |
| Burger M. N. | 2022 | Experimental study | RoB 2 | Some concerns |
| Buttenheim | 2020 | RCT | RoB 2 | Low risk |
| Chiavenna C. | 2023 | RCT | RoB 2 | Some concerns |
| Cole J.W. | 2022 | Before–after / non-randomized study | ROBINS-I | Moderate |
| Cunningham R.M. | 2021 | RCT | RoB 2 | Some concerns |
| Dai H. | 2021 | RCT | RoB 2 | Some concerns |
| Daley M.F. | 2018 | RCT | RoB 2 | Some concerns |
| Debroy P | 2023 | RCT | RoB 2 | High risk |
| Eitze S. | 2021 | Experimental study | RoB 2 | High risk |
| Fisher K.A. | 2023 | RCT | RoB 2 | Some concerns |
| Fishman J. | 2022 | RCT | RoB 2 | Some concerns |
| Gagneur A. | 2018 | Before–after / non-randomized study | ROBINS-I | Low |
| Gagneur A. | 2019 | RCT | RoB 2 | High risk |
| Galasso V. | 2023 | Experimental study | RoB 2 | Some concerns |
| Glanz J.M. | 2020 | RCT | RoB 2 | Low risk |
| Henrikson N.B. | 2015 | RCT | RoB 2 | Low risk |
| Holford, D. | 2024 | RCT | RoB 2 | Some concerns |
| Humlum, M.K. | 2021 | Experimental study | RoB 2 | Some concerns |
| Jacobson | 2022 | RCT | RoB 2 | High risk |
| Jamison K.C. | 2022 | Experimental study | RoB 2 | Some concerns |
| Jimenez A.V. | 2018 | Experimental study | RoB 2 | High risk |
| Joslyn S. | 2023 | RCT | RoB 2 | Low risk |
| Kerr, J.R. | 2021 | RCT | RoB 2 | Some concerns |
| La Torre, G. | 2020 | Experimental study | RoB 2 | Low risk |
| Lewin, E.B. | 2024 | RCT | RoB 2 | Some concerns |
| Mäki K.O. | 2023 | RCT | RoB 2 | High risk |
| Mills F. | 2023 | RCT | RoB 2 | High risk |
| Opel D.J. | 2019 | RCT | RoB 2 | Low risk |
| Panozzo C.A. | 2020 | RCT | RoB 2 | Some concerns |
| Peters E. | 2024 | RCT | RoB 2 | High risk |
| Pfattheicher S. | 2022 | RCT | RoB 2 | High risk |
| Piltch-Loeb R. | 2022 | Experimental study | RoB 2 | High risk |
| Real F.J. | 2017 | RCT | RoB 2 | Some concerns |
| Reno J.E. | 2019 | RCT | RoB 2 | Some concerns |
| Robertson D.A. | 2022 | RCT | RoB 2 | Some concerns |
| Rodriguez R.M. | 2023 | RCT | RoB 2 | Some concerns |
| Ronzani P | 2022 | Experimental study | RoB 2 | Some concerns |
| Sääksvuori L. | 2022 | RCT | RoB 2 | Some concerns |
| Schneider F. H. | 2023 | RCT | RoB 2 | Some concerns |
| Sudharsanan N. | 2022 | RCT | RoB 2 | Low risk |
| Szaszi, A.J. | 2024 | RCT | RoB 2 | Low risk |
| Szilagyi P.G. | 2023 | RCT | RoB 2 | Low risk |
| Takagi M.A. | 2023 | Experimental study | RoB 2 | Low risk |
| Teličák P. | 2024 | Experimental study | RoB 2 | Low risk |
| Vandeweerdt C. | 2022 | RCT | RoB 2 | Some concerns |
| Vaughn A.R. | 2018 | Experimental study | RoB 2 | Low risk |
| Verger P. | 2023 | RCT | RoB 2 | Low risk |
| Williams, S.E. | 2013 | RCT | RoB 2 | Low risk |
| Witus, L. | 2022 | RCT | RoB 2 | Low risk |
| Yousuf H. | 2021 | RCT | RoB 2 | Low risk |
| Zapf A.J. | 2024 | RCT | RoB 2 | Some concerns |
| Zhu P. | 2022 | RCT | RoB 2 | High risk |

Supplementary Figure 1. Overall risk of bias assessment of the included studies. Interventions addressing vaccine hesitancy in the WHO European Region and North America (United States and Canada), 2013–2024.
